# Lipoprotein(a) and Cardiovascular Disease: From Genetic Risk Factor to Therapeutic Target

**DOI:** 10.3390/cells15040315

**Published:** 2026-02-07

**Authors:** Hyeong Rok Yun, Manish Kumar Singh, Sunhee Han, Jyotsna S. Ranbhise, Joohun Ha, Sung Soo Kim, Insug Kang

**Affiliations:** 1 Department of Biochemistry and Molecular Biology, School of Medicine, Kyung Hee University, Seoul 02447, Republic of Korea; foryou018@naver.com (H.R.Y.);; 2 Biomedical Science Institute, Kyung Hee University, Seoul 02447, Republic of Korea; 3 Department of Biomedical Science, Graduate School, Kyung Hee University, Seoul 02447, Republic of Korea

**Keywords:** Lipoprotein(a), Apolipoprotein(a), atherosclerotic cardiovascular disease, calcific aortic valve stenosis

## Abstract

**Highlights:**

**What are the main findings?**

- Lipoprotein(a) [Lp(a)] acts as a distinct, genetically determined driver of cardiovascular disease by integrating pro-atherogenic, pro-inflammatory, and pro-thrombotic mechanisms.
- Emerging RNA-targeted platforms, such as antisense oligonucleotides and siRNAs, can achieve potent and durable Lp(a) reductions of over 90%, overcoming the limitations of conventional therapies.

**What are the implications of the main findings?**

- The development of potent Lp(a)-lowering therapeutics facilitates a transition from identifying Lp(a) as a risk marker to utilizing it as a specific therapeutic target.
- Ongoing Phase 3 clinical trials will define the clinical necessity of routine Lp(a) screening and the potential for mitigating residual risk in patients with ASCVD and aortic stenosis.

**Abstract:**

Lipoprotein(a) [Lp(a)] is a causal, genetically determined risk factor for atherosclerotic cardiovascular disease (ASCVD) and calcific aortic valve stenosis (CAVS). Although elevated Lp(a) affects approximately 20% of the global population, specific pharmacological options have long been unavailable, leaving a major gap in residual risk management. This review synthesizes current understanding of Lp(a) molecular architecture, genetics, and metabolism, and integrates mechanistic evidence linking Lp(a) to pro-atherogenic, pro-inflammatory, and pro-thrombotic pathways. We summarize epidemiological and genetic data associating Lp(a) with a broad spectrum of cardiovascular outcomes and discuss current clinical guidelines on screening and risk stratification. Furthermore, we provide an up-to-date overview of the emerging therapeutic landscape, including RNA-targeted therapies and novel oral small molecules. With pivotal phase 3 outcome trials nearing completion, the field is transitioning from viewing Lp(a) as an untreatable biomarker to an actionable therapeutic target, with important implications for precision cardiovascular prevention.

## 1. Introduction

Lipoprotein(a) [Lp(a)] is recognized as a distinct, genetically determined lipoprotein species that is established as an independent causal factor for both atherosclerotic cardiovascular disease (ASCVD) and calcific aortic valve stenosis (CAVS) [1,2,3]. Elevated Lp(a) affects approximately 20% of the global population [4,5]. While historically categorized merely as a marker of residual risk that persists despite optimal control of low-density lipoprotein cholesterol (LDL-C), contemporary evidence now establishes elevated Lp(a) as a distinct, primary driver of atherothrombosis and valvular pathology. Collectively, these data establish Lp(a) as an independent causal driver of ASCVD and CAVS. Importantly, its risk not simply ‘residual low-density lipoprotein (LDL) risk’ but is mediated through distinct pro-inflammatory, pro-thrombotic and pro-calcific pathways. Large-scale epidemiologic cohorts and Mendelian randomization studies have substantiated a robust, dose-dependent association between circulating Lp(a) concentrations and a diverse array of cardiovascular outcomes [6,7]. Mechanistically, this excess risk is attributable to the unique molecular architecture of Lp(a), comprising an LDL-like particle covalently linked to the highly polymorphic apolipoprotein(a), which exerts pro-atherogenic, pro-inflammatory and pro-thrombotic effects distinct from conventional LDL-driven dyslipidemia [8,9,10]. Current guidelines recommend universal, one-time Lp(a) screening in adults, classifying elevated concentrations as a risk-enhancing factor that necessitates aggressive risk modification [11,12]. Specific pharmacological treatments for elevated Lp(a) remain unavailable, with conventional lipid-lowering therapies and lifestyle modifications demonstrating negligible efficacy [13,14]. We stand on the verge of a paradigm shift in cardiovascular medicine. With the imminent completion of phase 3 cardiovascular outcome trials for RNA-targeted therapies and the advent of oral small molecules like muvalaplin, Lp(a) is transitioning from an untreatable genetic marker to a potent therapeutic target.

Here, we synthesize current knowledge regarding the pathobiology of Lp(a) and integrate clinical epidemiology and guideline recommendations, while critically evaluating the emerging landscape of novel Lp(a)-targeted therapeutics.

## 2. Structure, Genetics and Metabolism of Lp(a)

### 2.1. Unique Molecular Architecture

**Figure 1:**
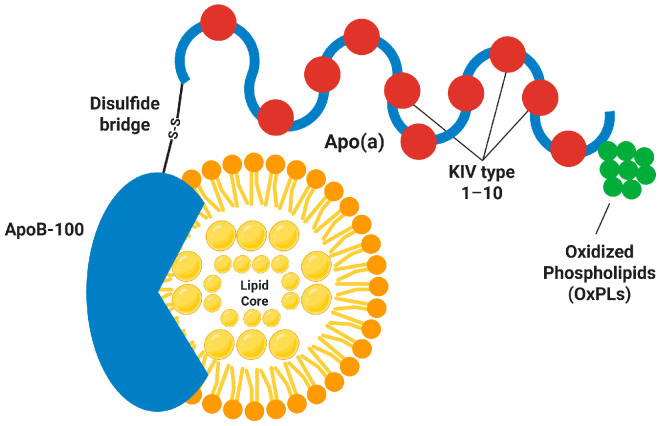
Molecular architecture of Lipoprotein(a). The schematic illustrates the unique bipartite structure of Lp(a), comprising an LDL-like particle containing apolipoprotein B-100 (apoB-100) covalently linked to apolipoprotein(a) [apo(a)] via a single disulfide bridge. Apo(a) contains multiple Kringle domains, resembling a bead-on-a-string structure. The red dots represent oxidized phospholipids (OxPLs), which are covalently attached to apo(a) and mediate pro-inflammatory effects. (Figure created in Biorender. Hyeong Rok Yun. (2025) https://app.biorender.com/illustrations/canvas-beta/6957731e86a383a1f4bf115. Accessed on 5 February 2026).

Lp(a) is characterized by a distinct bipartite molecular architecture, comprising an LDL-like moiety and a hydrophilic glycoprotein, apolipoprotein(a) [apo(a)]. The lipid core is structurally analogous to that of LDL, consisting of a hydrophobic center rich in cholesteryl esters and triglycerides, encased by a phospholipid and unesterified cholesterol monolayer, and scaffolded by a single molecule of apolipoprotein B-100 (apoB-100). The hallmark of Lp(a) is the covalent linkage between apo(a) and apoB-100 via a single disulfide bridge connecting the Cys4326 residue of apoB-100 to the Cys4057 residue within the Kringle IV type 9 (KIV-9) domain of apo(a). This distinct covalent coupling distinguishes Lp(a) from all other lipoprotein classes and contributes to its distinct metabolic properties [15,16]. The detailed molecular architecture of Lp(a), highlighting the covalent linkage between apoB-100 and the multi-kringle structure of apo(a) decorated with oxidized phospholipids, is schematically presented in Figure 1. Apo(a) is highly homologous to plasminogen, the principal fibrinolytic zymogen, but exhibits a distinct domain architecture [17,18]. Plasminogen comprises five distinct Kringle domains (KI–KV) and a proteolytic domain, whereas apo(a) contains ten distinct Kringle IV (KIV) types, a single Kringle V domain, and a protease-like domain [19]. Critically, the KIV type 2 (KIV-2) domain is characterized by a highly variable number of identical repeats, ranging from approximately 2 to >40 copies per allele. This variation underlies the substantial size heterogeneity of apo(a), which spans a molecular mass of 300–800 kDa [20,21,22]. Notably, although the protease-like domain is structurally homologous to the catalytic domain of plasminogen, key amino acid substitutions render the domain catalytically inert. Consequently, Lp(a) lacks intrinsic fibrinolytic activity while competing with plasminogen for binding sites and attenuating plasminogen [9,18]. Crucially, Lp(a) constitutes the major physiological carrier of oxidized phospholipids (OxPLs) in human plasma [8]. These OxPLs are primarily sequestered within KIV type 10 (KIV-10) domain of apo(a), reflecting a stable covalent attachment to lysine residues via Schiff base formation. While this covalent linkage modifies the OxPL molecule, it ensures that Lp(a) serves as a protected reservoir, effectively delivering these pro-inflammatory mediators to the vascular wall while shielding them from degradation. These pro-inflammatory lipids are covalently linked to apo(a), predominantly via the KIV-10 domain, and are sequestered within the lipid phase. Lysine-binding sites within the Kringle domains facilitate Lp(a) interactions with fibrin, cell surface receptors and extracellular matrix components, thereby underpinning its pathogenicity as a carrier of pro-inflammatory mediators [23,24,25,26].

### 2.2. Genetics and Isoform Size Heterogeneity

Circulating Lp(a) concentrations exhibit exceptionally high heritability, with >90% of the inter-individual variance in plasma levels attributable to genetic variation at the LPA gene locus on chromosome 6q26–27 [27,28]. The principal genetic determinant driving this variability is the copy number variation (CNV) within the KIV-2 encoding region [29]. Plasma Lp(a) concentration exhibits a robust inverse log-linear correlation with the number of KIV-2 repeats, and consequently, with the molecular mass of the apo(a) isoform [30,31]. Hepatocytes secrete smaller isoforms efficiently, driven by rapid folding kinetics. In contrast, larger isoforms are impeded by extensive quality control surveillance in the endoplasmic reticulum (ER), leading to significant intracellular turnover prior to secretion [32,33,34]. Consequently, alleles encoding smaller apo(a) isoforms are generally associated with lifelong elevations in plasma Lp(a) [31,35,36]. In addition to KIV-2 CNV, specific single-nucleotide polymorphisms (SNPs) such as rs10455872 and rs3798220, serve as robust genetic surrogates for small apo(a) isoforms. These variants are strongly implicated in both marked elevations of plasma Lp(a) and heightened cardiovascular risk [1,12,37,38].

The distribution of Lp(a) levels and isoform sizes exhibits marked heterogeneity across diverse ancestries. Populations of European and East Asian descent, including Korean, Japanese, and Chinese cohorts, typically display a positively skewed distribution characterized by lower median concentrations but a prominent rightward tail representing individuals with extreme elevations. While the inverse correlation between isoform size and plasma concentration is universally conserved across diverse ancestries, individuals of African descent consistently exhibit higher Lp(a) levels relative to isoform size. This observation implicates the contribution of ancestry-specific cis-regulatory variants or promoter modifications that operate independently of KIV-2 copy number [18,36,39,40,41].

### 2.3. Metabolism: Synthesis and Clearance

Lp(a) metabolism differs fundamentally from that of LDL. Apo(a) is synthesized exclusively in hepatocytes, whereas assembly of the mature Lp(a) particle occurs extracellularly, likely at or near the hepatocyte surface [42,43]. In contrast to LDL, whose clearance is largely mediated by the LDL receptor (LDLR), the catabolic pathways responsible for Lp(a) removal remain incompletely defined [42]. Available human kinetic and genetic evidence supports, at most, a minor contribution of LDLR to Lp(a) clearance, providing a mechanistic explanation for why therapies that upregulate LDLR expression have limited efficacy in lowering Lp(a) [14,44]. Accordingly, attention has shifted to alternative pathways, including plasminogen receptor-related mechanisms [e.g., plasminogen receptor with a C-terminal lysine (PlgRKT)], scavenger receptor class B type I (SR-BI), and renal handling of apo(a) fragments [44,45,46,47]. These distinctions underscore the need for Lp(a)-directed therapies that act independently of canonical LDLR-mediated pathways.

## 3. Pathologic Mechanisms Between Lp(a) and Cardiovascular Disease

Lp(a) promotes cardiovascular disease through convergent pro-atherogenic, pro-inflammatory, pro-thrombotic and pro-calcific mechanisms that span the arterial wall, circulating compartment and aortic valve [48,49,50]. In the current paradigm, Lp(a) is not simply a passive apoB-containing cholesterol carrier but acts as a genetically determined lipoprotein platform that preferentially transports oxidized phospholipids and other bioactive cargos into disease-relevant vascular and valvular microenvironments. This targeted delivery amplifies chronic lesion initiation and progression and can potentiate acute clinical events, thereby positioning Lp(a) as a key mediator of residual cardiometabolic risk and calcific valvular pathology [10,51,52,53,54]. At the level of the arterial wall, the LDL-like moiety of Lp(a) infiltrates the endothelium and accumulates within the arterial intima in a manner broadly similar to other apoB-containing lipoproteins, while exhibiting distinct physicochemical and molecular features that promote prolonged intimal retention [46,55,56]. Electrostatic interactions with negatively charged proteoglycans and components of the extracellular matrix mediate the avid binding and persistence of Lp(a) within the subendothelial compartment [57,58]. Upon prolonged subendothelial residence, Lp(a) is subject to oxidative and enzymatic modifications that enhance recognition by macrophage scavenger receptors, thereby promoting foam cell formation and facilitating progression from early fatty streaks to more advanced atherosclerotic lesions [59,60]. In parallel, the apo(a) moiety exhibits high affinity for extracellular matrix proteins and stimulates smooth muscle cell proliferation and migration, thereby contributing to fibrous cap development and maladaptive arterial remodeling [61,62]. Collectively, these lesion-structuring mechanisms increase both the quantitative burden and qualitative complexity of atherosclerotic plaques in a manner that is, at least in part, independent of LDL-C [48,63].

As a major carrier of oxidized phospholipids in the circulation, Lp(a) promotes endothelial activation, characterized by reduced nitric oxide bioavailability and upregulation of adhesion molecules such as vascular cell adhesion molecule-1 (VCAM-1), intercellular adhesion molecule-1 (ICAM-1) and E-selectin, thereby promoting leukocyte rolling, firm adhesion, and transendothelial migration [51,64,65,66]. In monocytes and macrophages, Lp(a) and its oxidized phospholipids engage pattern-recognition and lipid-sensing receptors including Toll-like receptors and scavenger receptors, thereby activating nuclear factor kappa B (NF-κB) signaling and the NLR family pyrin domain containing 3 (NLRP3) inflammasome and increasing the secretion of interleukin-1β, interleukin-6, tumor necrosis factor-α and chemokines such as monocyte chemoattractant protein-1 (MCP-1). This program sustains chronic, low-grade vascular inflammation and is associated with accelerated plaque progression and features of plaque vulnerability [64,67,68].

Within the hemostatic system, Lp(a), via structural homology of apo(a) to plasminogen, competes with plasminogen for fibrin binding sites without supporting fibrinolysis. This competitive interference attenuates plasmin generation, impairs tissue plasminogen activator-dependent fibrinolysis and prolongs the persistence of fibrin-rich thrombi at sites of plaque disruption [69,70,71,72]. Recent experimental evidence suggests that non-oxidized Lp(a), particularly at lower concentration, has limited effects on fibrinolysis. In contrast, highly oxidized Lp(a) more potently inhibits plasminogen activation and is associated with a pronounced pro-thrombotic phenotype [12,73,74]. Beyond competitive effects on plasminogen, Lp(a) enhances tissue factor expression, increases platelet activation and interacts with components of the complement system, thereby shifting the hemostatic balance toward thrombosis. Consequently, thrombus formation after plaque rupture or erosion is more likely to become flow-limiting or occlusive [10,49,73].

Lp(a) is also implicated in the pathogenesis of calcific aortic valve disease. Lp(a) and its associated oxidized phospholipids accumulate within the aortic valve leaflets and are internalized by valvular endothelial cells (VECs) and valvular interstitial cells (VICs) [75,76,77]. In VICs, Lp(a) signaling upregulates alkaline phosphatase activity and promotes calcium and phosphate deposition. This calcific process is driven by the activation of osteogenic transcriptional programs, involving runt-related transcription factor 2 (Runx2), bone morphogenetic protein 2 (BMP2) and canonical Wingless/Int-1 (Wnt)/β-catenin pathways, as well as p38 mitogen-activated protein kinase (MAPK) and glycogen synthase kinase 3 beta (GSK3β) signaling [77,78,79]. Collectively, these phenotypic shifts promote the formation of calcific nodules and progressive valvular stiffening. In parallel, Lp(a) induces the production of interleukin-6, interleukin-8, MCP-1 and other mediators, thereby recruiting inflammatory cells and stimulating fibrotic extracellular matrix accumulation. Accordingly, inflammation, fibrosis and calcification evolve as an interconnected continuum that culminates in leaflet thickening and restricted leaflet motion [80,81,82]. Recent evidence suggests that Lp(a) can reprogram metabolic pathways in VICs, shifting substrate utilization toward a state that supports osteogenic differentiation and sustained calcification. These observations further suggest that Lp(a) may impose a persistent metabolic imprint on the valve microenvironment [82,83,84].

The deleterious impact of Lp(a) is further compounded by its complex interactions with established risk factors and comorbidities. Elevated Lp(a) levels are consistently associated with higher rates of myocardial infarction and other manifestations of atherosclerotic cardiovascular disease [85,86,87]. Notably, in patients receiving intensive LDL-C-lowering therapy, Lp(a) frequently emerges as a substantial driver of residual atherosclerotic risk. In insulin resistance and type 2 diabetes, concomitant elevation of Lp(a) together with systemic inflammation and metabolic dysfunction appears to drive particularly adverse clinical outcomes [83,88,89,90,91]. This observation is consistent with the paradigm that metabolic and inflammatory axes potentiate Lp(a)-mediated vascular injury. In chronic kidney disease, reduced Lp(a) catabolism, increased oxidative stress and alterations in lipoprotein composition may increase the oxidized phospholipid load and thereby intensify the risk associated with specific Lp(a) concentrations [52,92,93,94]. Collectively, these findings support the paradigm that Lp(a) is not simply an isolated risk marker, but a risk amplifier, with its clinical impact shaped by the broader cardiometabolic and renal milieu.

Several conceptual questions remain under active investigations. A central issue concerns the relative contribution of “quantity” and “quality,” namely whether cardiovascular risk is driven predominately by absolute Lp(a) concentration or by qualitative attributes such as oxidized phospholipid content, apo(a) isoform size, glycation and other post-translational modifications [52,88,95]. Another question concerns the relative contribution of lesion-structuring atherogenic mechanisms versus inflammatory and immunologic pathways. Contemporary reviews increasingly emphasize the integrated innate immune framework in which Lp(a) functions as a lipid-immune complex that couples apoB-driven retention with oxidized phospholipid-dependent immune activation [49,67,96]. Furthermore, there is ongoing debate regarding the potential dualistic or context-dependent modulation of fibrinolysis and thrombosis by Lp(a). Emerging evidence suggests that the net hemostatic phenotype is influenced by both circulating Lp(a) concentration and the biochemical modification of lipoprotein. Finally, it remains to be fully elucidated whether profound and sustained Lp(a) lowering achieved with antisense oligonucleotide or small interfering RNA therapeutics will modify plaque composition, influence the trajectory of aortic valve calcification, or affect microvascular and thrombotic phenotypes. The pivotal phase 3 cardiovascular outcome trials currently delineate the reversibility of these downstream pathways and define the proportion of Lp(a)-associated risk that can be mitigated in clinical practice.

## 4. Epidemiology and Clinical Associations

### 4.1. Coronary Artery Disease and Myocardial Infarction

A convergence of evidence from prospective cohort studies, case–control investigations, and Mendelian randomization analyses has established a robust, approximately log-linear association between circulating Lp(a) concentrations and the risk of coronary artery disease (CAD) and myocardial infarction (MI) [7,85]. In population-based cohorts such as EPIC-Norfolk and other large-scale investigations, individuals in the highest Lp(a) category consistently demonstrated a 2- to 3-fold increased risk of coronary artery disease (CAD) compared to those in the lowest category. Notably, this association remained robust even after comprehensive adjustment for LDL-C and other traditional risk factors [97,98]. Crucially, this relationship appears to be continuous, lacking a distinct threshold below which Lp(a) concentrations are entirely benign. Nevertheless, clinical guidelines commonly apply pragmatic cut points such as ≥50 mg/dL (≥125 nmol/L), to identify individuals in a high-risk category [99]. Recent data from a large-scale US cohort have extended the generalizability of these findings to contemporary populations, encompassing patients with diabetes and those receiving standard-of-care preventive therapies. In these groups, elevated Lp(a) conferred an independent increase in long-term risk of atherosclerotic cardiovascular disease (ASCVD), with risk gradients preserved across strata defined by LDL-C levels and irrespective of statin utilization. These findings support the role of Lp(a) as a determinant of risk despite guideline-directed LDL-lowering therapy [85,90]. In secondary prevention cohorts comprising patients with a history of MI, elevated Lp(a) is associated with a higher rate of recurrent events and major adverse cardiovascular events (MACE), notably during the initial years of follow-up. Ethnic- and region-specific studies further support the generalizability of these associations [86,100]. Collectively, these findings indicate that Lp(a) represents a stable, largely lifelong determinant of coronary risk, with higher concentrations conferring progressively greater likelihood of MI across diverse populations and clinical landscape.

### 4.2. Ischemic Stroke and Peripheral Artery Disease

Beyond coronary artery disease, elevated Lp(a) constitutes a significant risk determinant for ischemic stroke, most consistently for large artery atherosclerotic stroke, and with a broader spectrum of peripheral vascular diseases [7,101]. In EPIC-Norfolk and other prospective cohorts, higher Lp(a) concentrations were associated with increased incidence of peripheral artery disease (PAD) and coronary outcomes. Association with ischemic stroke were more modest and heterogeneous across cohorts, but are supported in large contemporary datasets [102,103]. Recent investigations have further substantiated the causal relationship between Lp(a) and peripheral vascular pathology. A pivotal study integrating observational and genetic data demonstrated a two- to three-fold increase in the risk of PAD, abdominal aortic aneurysm, and major adverse limb events (MALEs) in individuals with elevated Lp(a) [104]. These findings support the concept that Lp(a) is an important contributor to systemic atherosclerotic disease, with vascular consequences extending beyond the coronary circulation. Narrative and systematic reviews examining PAD and carotid atherosclerosis have identified Lp(a) as a critical, yet frequently underrecognized, risk factor within these vascular territories [105,106]. Although select cohorts of patients with advanced, symptomatic PAD on intensive statin therapy have not demonstrated incremental prognostic value for Lp(a), this likely reflects a ceiling effect in ultra-high-risk populations or the predominance of competing risks, rather than a lack of causality. Overall, the epidemiologic evidence supports Lp(a) as a marker of generalized atherothrombotic burden extending to cerebrovascular and peripheral arterial beds, particularly in the context of primary prevention and early secondary prevention.

### 4.3. Calcific Aortic Valve Stenosis

Calcific aortic valve stenosis (CAVS) has emerged as one of the clinical phenotypes most strongly associated with elevated Lp(a). Convergent evidence from observational cohorts, genome-wide association studies (GWAS), and Mendelian randomization analyses supports a causal role for elevated Lp(a) concentrations and LPA risk variants in driving both the initiation and subsequent hemodynamic progression of aortic valve calcification and clinical stenosis, independent of LDL-C and conventional risk factors [107]. Meta-analyses indicate that individuals with elevated Lp(a) carry an approximately 1.5- to 2-fold increased risk of incident CAVS [108]. Furthermore, higher baseline Lp(a) levels are associated with accelerated hemodynamic progression once the disease is established [109]. Recent investigations have extended these observations from incidence to clinically meaningful endpoints. Notably, a 2025 analysis in Journal of the American Heart Association (JAHA) demonstrated that patients with CAVS and elevated Lp(a) experienced significantly higher rates of composite valvular and cardiovascular events, encompassing the need for aortic valve replacement (AVR) and hospitalization for heart failure [83]. Contemporary reviews increasingly characterize Lp(a) not merely as a marker, but as a central upstream mediator across the CAVS disease continuum, spanning from early aortic sclerosis to severe stenosis. These insights have catalyzed calls to incorporate Lp(a) assessment into risk stratification algorithms for patients with subclinical valve disease, although definitive guideline recommendations remain under active development.

### 4.4. Heart Failure and Long-Term Prognosis

The relationship between Lp(a) and heart failure (HF) is more complex and appears to be predominantly mediated by the cumulative burden of antecedent coronary atherosclerosis and valvular pathology. In community-based cohorts, elevated Lp(a) concentrations have been associated with increased risk of incident heart failure (HF). This association appears to be largely mediated by antecedent atherosclerotic and valvular disease, particularly myocardial infarction and aortic valve stenosis, and has been observed across EF subtypes in contemporary cohorts [110,111,112]. Elevated Lp(a) has also been associated with adverse HF outcomes in patients with established cardiovascular disease, although disentangling direct myocardial effects from the sequelae of antecedent MI and CAVS remains challenging [113]. Recent studies have begun to evaluate the incremental prognostic utility of Lp(a) in multi-variable risk models. In real-world clinical cohorts, incorporation of Lp(a) alongside conventional risk factors enhances ASCVD risk prediction, including measures of discrimination and reclassification, particularly among individuals at intermediate baseline risk [114,115]. In patients with prior cardiovascular events, elevated Lp(a) concentrations identify subgroups at increased risk of early recurrence, suggesting potential utility for tailoring the intensity of follow-up and preventive therapy [86,100]. Conversely, within the specific domain of HF, evidence remains limited, it is uncertain to what extent Lp(a)-associated risk reflects direct myocardial or microvascular effects, as opposed to mediation through cumulative ischemic injury and valvular disease burden over time [110,112]. Collectively, epidemiologic data consistently demonstrate that Lp(a) represents a stable, genetically determined determinant of lifelong exposure, elevating the risk for diverse array of cardiovascular pathologies, including MI, ischemic stroke, PAD and CAVS, while also potentially implicating it in the pathogenesis and progression of HF. The magnitude and consistency of these associations across diverse populations and study designs provide a strong empirical premise for ongoing trials designed to test whether potent, specific lowering of Lp(a) confers a clinically significant reduction in cardiovascular outcomes.

## 5. Measurement and Clinical Interpretation

### 5.1. Analytical Considerations and Standardization

Accurate quantification of Lp(a) is complicated by the size heterogeneity of apo(a) [116]. Several commonly used clinical assays quantify Lp(a) as mass concentration (mg/dL) using antibodies directed against the KIV-2 repeat region [117]. Given that KIV- 2 copy number varies substantially between individuals, such assays exhibit significant isoform-dependent bias, tending to overestimate Lp(a) in larger isoforms and underestimate it in smaller ones [118]. Accordingly, expert consensus and laboratory guidance recommend isoform-insensitive immunoassays calibrated to World Health Organization (WHO)/International Federation of Clinical Chemistry and Laboratory Medicine (IFCC) reference material, with results reported in molar units (nmol/L) [12,119,120]. Molar concentration directly reflects circulating particle number—the principal determinant of atherothrombotic and calcific risk—whereas conversion between mass and molar units remains imprecise given the variable molecular mass of apo(a). The molecular mass of Lp(a) exhibits significantly variable, typically ranging from 300 KDa to 800 KDa, reflecting the extensive size heterogeneity of apo(a) isoforms. Such variation underscores the clinical advantage of expressing Lp(a) concentrations in molar units (nmol/L), as this approach provides a standardized measure of the total number of pathogenic particles rather than their cumulative mass.

### 5.2. Guideline Thresholds for Risk Assessment

Major international guidelines have established thresholds to define elevated Lp(a) and guide risk stratification. The 2019 European Society of Cardiology (ESC)/European Atherosclerosis Society (EAS) guidelines and the 2022 EAS consensus statement classify Lp(a) levels into three categories: normal (<30 mg/dL or <75 nmol/L), intermediate (30–50 mg/dL or 75–125 nmol/L), and elevated (>50 mg/dL or >125 nmol/L) [11]. In the 2019 American College of Cardiology (ACC)/American Heart Association (AHA) Primary Prevention guidelines, ≥50 mg/dL (or ≥125 nmol/L) is designated a risk enhancing factor that can favor reclassification and intensification of preventive therapy in patients near decision thresholds [121]. European guidance also supports broader testing: the 2019 ESC/EAS dyslipidemia guideline recommends considering Lp(a) measurement at least once in each adult’s lifetime, particularly to identify individuals with very high inherited levels, and the 2025 ESC/EAS focused update reiterates once-in-a-lifetime measurement and emphasizes its value for risk refinement around treatment decision thresholds [122].

### 5.3. Clinical Indications and Timing

Lp(a) concentrations are largely genetically determined and remain relatively stable across adulthood, and a single measurement is generally sufficient for lifetime risk assessment [123]. Repeat measurement is rarely required, except during major systemic perturbations known to alter Lp(a), including nephrotic syndrome, advanced kidney disease, and acute inflammatory states [120]. Measurement is particularly informative in selected clinical contexts, including premature ASCVD, commonly defined as myocardial infarction before 55 years in men and before 60 years in women, in the presence of only modest conventional risk factors, and recurrent atherosclerotic events despite intensive LDL cholesterol lowering [124]. Cascade screening is also recommended in families with a strong history of premature ASCVD or markedly elevated Lp(a) [125,126,127]. In addition, Lp(a) measurement is relevant in the evaluation of unexplained calcific aortic valve stenosis and in patients with accelerated, disproportionate multivessel atherosclerosis [109,128]. In primary prevention, Lp(a) measurement provides risk reclassification among individuals at borderline or intermediate estimated risk and helps individualize the intensity of long-term preventive strategies.

## 6. Current Management Strategies

### 6.1. Lifestyle and Conventional Lipid-Lowering Therapy

Lifestyle modifications including dietary optimization and regular physical activity constitute the foundation of global cardiovascular risk reduction. However, these measures exert negligible influence on circulating Lp(a) concentration [129]. Dietary changes and weight loss improve metabolic parameters such as blood pressure and systemic inflammation. Despite these benefits, such interventions fail to elicit clinically meaningful reductions in Lp(a) levels. Regarding pharmacotherapy, statins represent the cornerstone of LDL-C management. These agents exhibit no efficacy in lowering Lp(a) and may paradoxically induce a modest elevation in concentration. Statin therapy remains imperative due to substantial reduction in atherosclerotic cardiovascular disease risk via LDL-C lowering [129,130,131,132]. Ezetimibe also provides incremental LDL-C reduction in combination with statins. However, this agent exerts a neutral effect on Lp(a) levels [120]. Conversely, monoclonal antibodies against proprotein convertase subtilisin/kexin type 9 (PCSK9), such as evolocumab and alirocumab, demonstrate a consistent ability to lower Lp(a) by approximately 20 to 30 percent. This reduction occurs alongside profound decreases in LDL-C. Analyses of large outcome trials suggest that patients with elevated baseline Lp(a) derive pronounced risk reductions from PCSK9 inhibitors. Distinguishing the specific therapeutic benefit attributable to Lp(a) lowering from potent LDL-C reduction remains challenging [133,134]. Overall, currently available lipid lowering therapies should be regarded primarily as LDL cholesterol targeted interventions with modest and often incidental effects on Lp(a). Accordingly, management of patients with elevated Lp(a) should prioritize guideline-directed intensification of LDL cholesterol lowering rather than expectations of substantial Lp(a) reduction.

### 6.2. Lipoprotein Apheresis

Lipoprotein apheresis provides a more direct but invasive means of lowering Lp(a). This extracorporeal technique acutely removes apoB-containing lipoproteins, including LDL and Lp(a), from the circulation and can achieve immediate reductions in Lp(a) on the order of 60–70% per session [135,136]. Observational studies in patients with progressive ASCVD and markedly elevated Lp(a) despite maximal medical therapy suggest that long-term apheresis may slow the progression of coronary disease and reduce clinical events, but randomized controlled trial data are limited, and the procedure is costly, time-consuming and available only in specialized centers [137,138]. Consequently, lipoprotein apheresis is reserved for selected patients, typically those with very high Lp(a) and refractory, rapidly progressive ASCVD or familial hypercholesterolemia, and is unlikely to be scalable as a population-level approach to Lp(a) management.

### 6.3. Practical Approach in the Absence of Lp(a)-Targeted Drugs

In the current therapeutic landscape, Lp(a)-targeted therapies have not received regulatory approval, and clinical outcome benefits of isolated Lp(a) lowering remain unproven [120]. Accordingly, elevated Lp(a) should be treated as a risk-enhancing factor that reclassifies estimated ASCVD risk, particularly in individuals near treatment decision thresholds [139,140]. The core strategy is aggressive lowering of LDL cholesterol and apolipoprotein B with maximally tolerated statins as foundational therapy, followed by stepwise addition of ezetimibe and PCSK9 inhibitors in appropriate candidates to achieve guideline-recommended targets [120,141,142]. Although PCSK9 inhibitors modestly reduce Lp(a), their established event reduction is largely attributable to LDL-C lowering [143,144]. Comprehensive risk-factor optimization remains essential, including guideline-directed control of blood pressure, glycemic status, body weight and tobacco exposure, alongside sustained lifestyle intervention despite minimal effects on circulating Lp(a) [120]. Antithrombotic therapy should follow standard indications, with low-dose aspirin reserved for secondary prevention and selected primary prevention patients after individualized bleeding risk assessment rather than initiation driven solely by elevated Lp(a) [145,146]. In the setting of uncertain residual risk, coronary artery calcium scoring can refine risk assessment and support treatment intensification, while coronary computed tomography angiography or functional testing may be considered in selected cases [147,148]. Lipoprotein apheresis remains an option for highly selected patients with extreme Lp(a) and progressive ASCVD despite optimal medical therapy, and enrollment in clinical trials of emerging Lp(a)-lowering agents should be prioritized as available [149,150]. The distinguishing characteristics and clinical status of these current and emerging therapeutic strategies are summarized in Table 1.

## 7. Emerging Lp(a)-Targeted Therapies

### 7.1. Antisense Oligonucleotides: Pelacarsen

Antisense oligonucleotides (ASOs) targeting apo(a) synthesis represent the first class of agents specifically designed to lower Lp(a). Pelacarsen is a N-acetylgalactosamine (GalNAc)-conjugated antisense oligonucleotide that selectively binds LPA mRNA in hepatocytes, leading to degradation of the transcript and reduction in apo(a) production [151,152,153]. Phase 2 trials have demonstrated dose-dependent reductions in Lp(a) of up to approximately 80%, with acceptable safety and tolerability profiles over the duration studied. These studies also showed consistent lowering of oxidized phospholipids associated with Lp(a), suggesting that pelacarsen not only decreases particle concentration but also attenuates the oxidant and inflammatory cargo carried by Lp(a) [154,155]. The pivotal Lp(a)HORIZON trial is a large, event-driven phase 3 study enrolling patients with established ASCVD and elevated Lp(a), designed to test whether long-term pelacarsen therapy reduces major adverse cardiovascular events beyond standard of care [156]. The results of this trial will be critical in determining whether selective antisense-mediated Lp(a) lowering translates into clinically meaningful risk reduction and will help define the magnitude of Lp(a) lowering required for benefit.

### 7.2. Small Interfering RNA: Olpasiran

**Figure 2:**
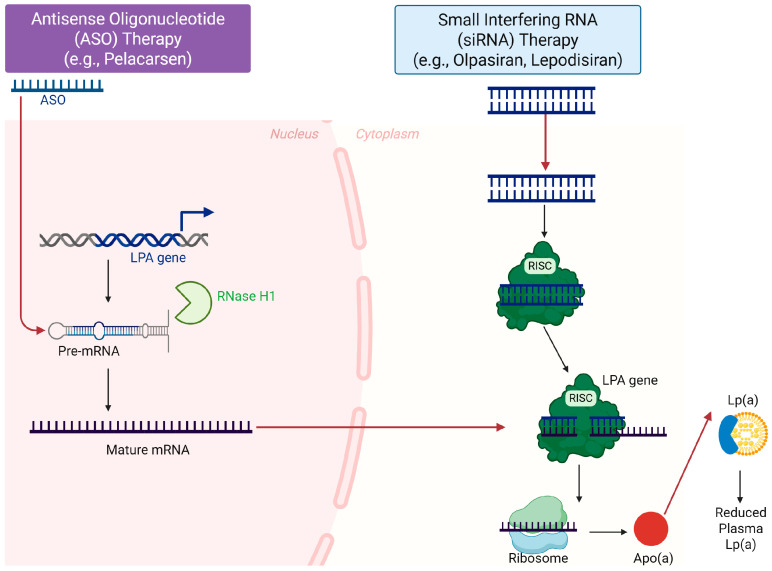
Mechanisms of action for emerging RNA-targeted Lp(a)-lowering therapeutics within a hepatocyte. This diagram illustrates how novel therapies inhibit apolipoprotein(a) synthesis inside a liver cell. Left: Antisense oligonucleotides (ASOs) like pelacarsen enter the nucleus and bind to *LPA* pre-mRNA. This recruits RNase H1, which cleaves and degrades the pre-mRNA. Right: Small interfering RNAs (siRNAs) like olpasiran and lepodisiran enter the cytoplasm and are loaded into the RNA-induced silencing complex (RISC). The RISC complex then binds to and degrades mature *LPA* mRNA. Both approaches prevent the translation of mRNA into the apo(a) protein by ribosomes, leading to a substantial reduction in the assembly and secretion of mature Lp(a) particles into the plasma. (Figure created in Biorender. Hyeong Rok Yun. (2025) https://app.biorender.com/illustrations/canvas-beta/69577e6509d04973d3de3b76. Accessed on 5 February 2026).

Small interfering RNA (siRNA) therapeutics offer an alternative gene-silencing strategy to inhibit hepatic apo(a) synthesis. Olpasiran is a GalNAc-conjugated siRNA directed against LPA mRNA and has shown striking potency in early-phase clinical studies [140,157]. In the phase 2 OCEAN(a)-DOSE trial, subcutaneous olpasiran administered at doses of 75 mg or higher every 12 weeks produced placebo-adjusted reductions in Lp(a) exceeding 95%, and the majority of treated patients achieved Lp(a) concentrations below 20 nmol/L, levels rarely observed in the general population [154,158]. Extension data indicate that substantial suppression of Lp(a) persists for many months after the last dose, suggesting that infrequent dosing may be feasible in long-term practice [140]. Safety data to date are encouraging, with mainly mild injection-site reactions and no clear safety signals, although larger and longer-term studies are required. The ongoing OCEAN(a)-Outcomes trial is evaluating whether olpasiran-induced Lp(a) lowering reduces coronary heart disease death, myocardial infarction and urgent coronary revascularization in patients with ASCVD and elevated Lp(a) [5,159]. Together with Lp(a)HORIZON, this trial will clarify not only the efficacy and safety of siRNA-mediated Lp(a) suppression but also the degree to which large and durable reductions in Lp(a) translate into fewer clinical events. The distinct subcellular mechanisms of action for these emerging RNA-targeted platforms, distinguishing between RNase H1-mediated nuclear degradation by ASOs and RISC-mediated cytoplasmic silencing by siRNAs, are depicted in Figure 2.

### 7.3. Lepodisiran and Other Emerging Agents

Lepodisiran, another GalNAc-conjugated siRNA targeting LPA, has shown similarly profound and remarkably durable Lp(a)-lowering effects in early-phase trials. Single or infrequent injections produced sustained reductions in Lp(a) of approximately 90% or more over many months [139,160]. These pharmacodynamic characteristics raise the possibility that Lp(a) could be controlled with very low-frequency dosing schedules, potentially improving adherence and reducing treatment burden if long-term safety is confirmed. In addition to pelacarsen, olpasiran and lepodisiran, several other agents are advancing through clinical development. SLN360 is an siRNA directed against LPA that has shown robust Lp(a) reductions in phase 1 and 2 studies, and additional antisense oligonucleotides are being optimized for potency, safety and dosing convenience [141,161,162]. While these RNA-targeted therapies have set a new benchmark for potency, the development of muvalaplin represents a pivotal innovation in therapeutic accessibility. Unlike gene-silencing agents that inhibit hepatic synthesis, muvalaplin is a first-in-class orally bioavailable small molecule that directly disrupts the electrostatic interaction between apo(a) and apoB-100, thereby preventing the assembly of the Lp(a) particle. This distinct mechanism allows for the lowering of Lp(a) without affecting the production of apoB or LDL-C [142,163,164]. By offering a non-injectable alternative, oral small molecules like muvalaplin hold the potential to broaden the therapeutic landscape, potentially improving long-term adherence and offering a complementary option to injectable biologics in the era of precision medicine.

## 8. Knowledge Gaps and Future Directions

Despite substantial progress in understanding Lp(a) biology and its clinical consequences, several critical knowledge gaps remain and will determine how Lp(a) is ultimately incorporated into cardiovascular prevention strategies. First, the quantitative relationship between the magnitude of Lp(a) reduction and clinical benefit is unknown: it is not yet clear how much Lp(a) must be lowered, and for how long, to achieve a clinically meaningful reduction in different types of events, or whether there is a concentration threshold below which further lowering yields diminishing returns. Second, the optimal timing of intervention over the life course has not been defined; given that Lp(a) is elevated from early childhood in affected individuals, it is uncertain whether the greatest absolute and relative benefits will accrue in secondary prevention, in high-risk primary prevention, or potentially with very early intervention before substantial atherosclerotic or valvular damage has occurred. Third, there is considerable uncertainty regarding heterogeneity of treatment effect, including how sex, ethnicity, LPA genotype, apo(a) isoform size, and comorbid conditions such as diabetes, chronic kidney disease, and systemic inflammatory disorders modify both the risk associated with elevated Lp(a) and the response to Lp(a)-lowering therapies. Furthermore, the potential influence of elevated Lp(a) on HDL metabolism remains an area of active investigation, reflecting a broader need to understand how high OxPL loads affect HDL functionality. While it is hypothesized that excessive OxPL may trigger oxidative modification of ApoA-I and subsequently impair the anti-oxidative capacity of HDL, definitive clinical evidence confirming this mechanism and its impact on HDL function remains to be established. Fourth, the extent to which Lp(a) lowering will influence non-atherosclerotic phenotypes—particularly the incidence and progression of calcific aortic valve stenosis, microvascular ischemia, and different heart failure subtypes—remains speculative and requires dedicated endpoints and imaging substudies in ongoing and future trials. In parallel, more detailed mechanistic work is needed to clarify how rapidly plaque composition, valvular calcification, and thrombotic and inflammatory biomarkers respond to large and sustained reductions in Lp(a), and whether any of the structural changes induced by decades of exposure are fully reversible. Additional priorities include the refinement of assays and units for Lp(a) measurement, standardization of risk thresholds across populations, integration of Lp(a) into multivariable risk prediction tools, and the development of pragmatic algorithms for identifying candidates for Lp(a)-targeted therapy in routine practice. Ultimately, the results of large phase 3 outcome trials with antisense and siRNA-based agents will be pivotal in resolving these uncertainties, determining the modifiability of Lp(a)-related risk, and defining whether Lp(a) should be treated primarily as a marker of inherited susceptibility or as a central, actionable therapeutic target in cardiovascular medicine [159].

## 9. Conclusions

Lipoprotein(a) represents a prevalent and potent genetic driver of residual cardiovascular risk and CAVS, distinguished by its unique convergence of pro-atherogenic, pro-inflammatory, and pro-thrombotic mechanisms. While clinical management has historically been limited to non-specific risk reduction, the landscape is rapidly shifting with the advent of RNA-based therapeutics capable of achieving profound and sustained reductions in Lp(a) levels. As results from large-scale outcome trials become available, the position of Lp(a) lowering within contemporary prevention strategies will be more clearly defined. Ultimately, the broader integration of Lp(a) measurement into routine clinical practice, combined with the successful deployment of these targeted therapies, holds the promise of significantly reducing the global burden of cardiovascular disease in the coming era of precision medicine.

## Figures and Tables

**Table 1:** Current and Emerging Therapeutic Strategies for Patient with Elevated Lp(a).

Strategy	Specific Intervention	Effect on Lp(a)	Clinical Status
Conventional	Statins/ Ezetimibe	Neutral/Slight increase	Standard of care for LDL-C
Established	PCSK9 Inhibitors	20~30% reduction	Approval for ASCVD/FH
Emerging (RNA)	Lipoprotein Apheresis	60~80% (acute)	Reserved for refractory cases
	Pelacarsen (ASO)	70~80% reduction	Phase 3 [Lp(a)HORIZON]
	Olpasiran (siRNA)	>90% reduction	Phase 3 [OCEAN(a) outcome]
	Muvalaplin (Small molecule)	65~90% reduction	Phase 2 (Oral)

## Data Availability

No new data were created or analyzed in this study.

## Abbreviations

The following abbreviations are used in this manuscript:

ACC	American College of Cardiology
AHA	American Heart Association
apo(a)	Apolipoprotein(a)
apoB-100	Apolipoprotein B-100
ASCVD	Atherosclerotic cardiovascular disease
ASO(s)	Antisense oligonucleotide(s)
AVR	Aortic valve replacement
BMP2	Bone morphogenetic protein 2
CAD	Coronary artery disease
CAVS	Calcific aortic valve stenosis
CNV	Copy number variation
EAS	European Atherosclerosis Society
EF	Ejection fraction
ER	Endoplasmic reticulum
ESC	European Society of Cardiology
GalNAc	N-acetylgalactosamine
GSK3β	Glycogen synthase kinase 3 beta
GWAS	Genome-wide association study
HF	Heart failure
ICAM-1	Intercellular adhesion molecule-1
IFCC	International Federation of Clinical Chemistry and Laboratory Medicine
IL-1β	Interleukin-1 beta
IL-6	Interleukin-6
JAHA	Journal of the American Heart Association
KI–KV	Kringle I–Kringle V
KIV	Kringle IV
KIV-10	Kringle IV type 10
KIV-2	Kringle IV type 2
KIV-9	Kringle IV type 9
LDL	Low-density lipoprotein
LDL-C	Low-density lipoprotein cholesterol
LDLR	Low-density lipoprotein receptor
Lp(a)	Lipoprotein(a)
LPA	LPA gene (apolipoprotein(a) gene locus)
MACE	Major adverse cardiovascular events
MALE(s)	Major adverse limb event(s)
MAPK	Mitogen-activated protein kinase
MCP-1	Monocyte chemoattractant protein-1
MI	Myocardial infarction
NF-κB	Nuclear factor kappa B
NLA	National Lipid Association
NLRP3	NLR family pyrin domain containing 3 (inflammasome)
OxPL(s)	Oxidized phospholipid(s)
PAD	Peripheral artery disease
PCSK9	Proprotein convertase subtilisin/kexin type 9
PlgRKT	Plasminogen receptor with a C-terminal lysine (PlgRKT)
RISC	RNA-induced silencing complex
Runx2	Runt-related transcription factor 2
siRNA	Small interfering RNA
SNP(s)	Single-nucleotide polymorphism(s)
SR-BI	Scavenger receptor class B type I
TNF-α	Tumor necrosis factor-alpha
tPA	Tissue plasminogen activator
VCAM-1	Vascular cell adhesion molecule-1
VEC(s)	Valvular endothelial cell(s)
VIC(s)	Valvular interstitial cell(s)
WHO	World Health Organization
Wnt	Wingless/Int-1 signaling pathway
